# Synthesis of the extended phenacene molecules, [10]phenacene and [11]phenacene, and their performance in a field-effect transistor

**DOI:** 10.1038/s41598-019-39899-4

**Published:** 2019-03-08

**Authors:** Hideki Okamoto, Shino Hamao, Ritsuko Eguchi, Hidenori Goto, Yasuhiro Takabayashi, Paul Yu-Hsiang Yen, Luo Uei Liang, Chia-Wei Chou, Germar Hoffmann, Shin Gohda, Hisako Sugino, Yen-Fa Liao, Hirofumi Ishii, Yoshihiro Kubozono

**Affiliations:** 1 Department of Chemistry, Okayama University, Okayama, 700-8530 Japan; 2 Research Institute for Interdisciplinary Science, Okayama University, Okayama, 700-8530 Japan; 3 Department of Physics, National Tsing Hua University, Hsinchu, 30013 Taiwan; 4 NARD Co Ltd, Amagasaki, 660-0805 Japan; 5 National Synchrotron Radiation Center, Hsinchu, 30076 Taiwan

## Abstract

The [10]phenacene and [11]phenacene molecules have been synthesized using a simple repetition of Wittig reactions followed by photocyclization. Sufficient amounts of [10]phenacene and [11]phenacene were obtained, and thin-film FETs using these molecules have been fabricated with SiO_2_ and ionic liquid gate dielectrics. These FETs operated in p-channel. The averaged measurements of field-effect mobility, <*μ*>, were 3.1(7) × 10^−2^ and 1.11(4) × 10^−1^ cm^2^ V^−1^ s^−1^, respectively, for [10]phenacene and [11]phenacene thin-film FETs with SiO_2_ gate dielectrics. Furthermore, [10]phenacene and [11]phenacene thin-film electric-double-layer (EDL) FETs with ionic liquid showed low-voltage p-channel FET properties, with <*μ*> values of 3(1) and 1(1) cm^2^ V^−1^ s^−1^, respectively. This study also discusses the future utility of the extremely extended π-network molecules [10]phenacene and [11]phenacene as the active layer of FET devices, based on the experimental results obtained.

## Introduction

The chemistry and physics of extended π-network molecules such as fullerene, graphite/graphene, and conjugated organic polymers/hydrocarbons are currently one of the most interesting and challenging frontiers of fundamental science, because these molecules have displayed excellent performance in electronics applications, as well as novel physical properties such as superconductivity^1–28^. The study of electronics using conjugated organic molecules has made rapid progress, in particular in electronics such as field-effect transistors (FETs) and solar cells^9–11,13–16,18–23,26–28^.

Our study of the fabrication and characterization of FETs using phenacene molecules, which consist of coplanar fused benzene rings in a repeating W-shaped pattern, has demonstrated excellent FET properties^19,23,26–28^. This high performance is due to the strong π-π interaction between phenacene molecules enabled by the extension of the benzene network. Showing a clear pattern, the field-effect mobility, *μ*, which is an indication of FET performance, has increased with the number of benzene rings, n; we have already synthesized phenacene molecules with 5–9 benzene rings, *i.e*., picene to [9]phenacene^19,26–28^. Thus the next step was to synthesize phenacene molecules with more than 9 benzene rings, and to fabricate FETs with the molecules. This was challenging because of the difficulty in synthesis caused by their quite low solubility in organic solvents; a low solubility often fatal in organic synthesis. Previously, only alkyl-substituted [11]phenacene^29,30^ and tetracarboxy [n]phenacene (n = 10, 12 and 14)^31^ had been synthesized, based for [n]phenacene molecules with n ≥ 10, but these are phenacene derivatives. No previous synthesis of unsubstituted phenacene molecules with n ≥ 10 has been reported, although they are quite significant as a matter of basic chemistry, as well as for their potential utility in electronic applications.

Here, we have synthesized the longest extended π-network molecules to date, [10]phenacene and [11]phenacene, using a simple repetition of Wittig reactions followed by photocyclization^29–35^. The synthesized samples were fully characterized using MALDI-time-of-flight mass spectrometry (MALDI-TOF MS), X-ray diffraction (XRD), and electronic absorption spectra, which confirmed the identity of these molecules. FET devices were then fabricated using thin films of these molecules, and their FET properties were fully investigated. The structural/electronic parameters of [10]phenacene and [11]phenacene, as well as their FET parameters, have been fully compared with those of other phenacene molecules, and the future prospect for FET application of extremely extended phenacene molecules is discussed.

## Results

### Preparation and characterization of [10]phenacene and [11]phenacene

**Figure 1:**
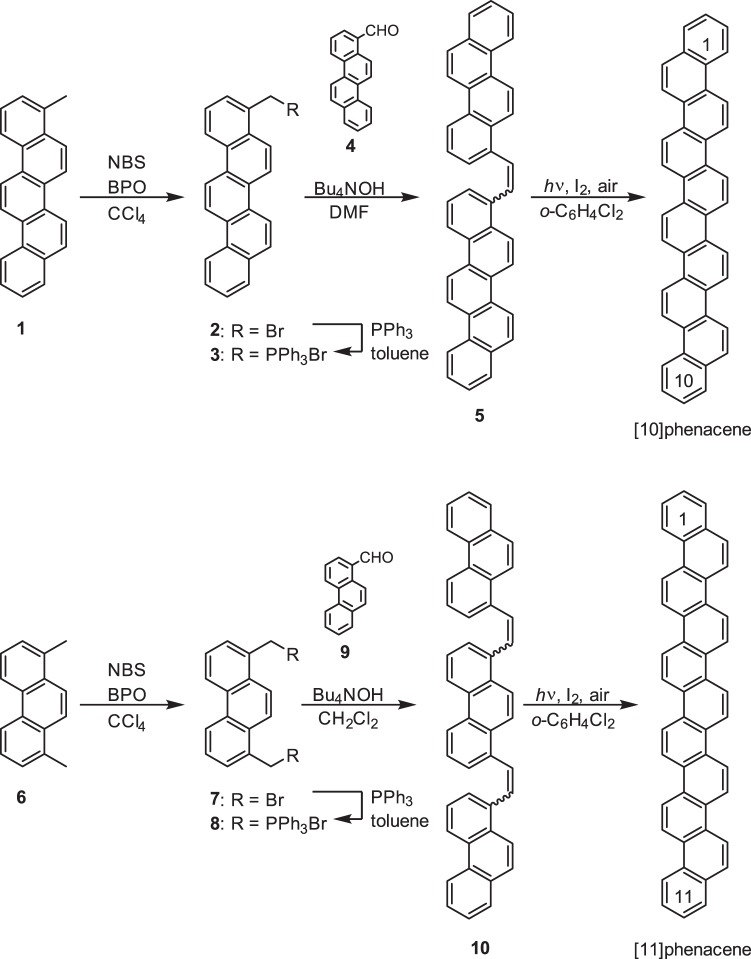
Synthetic routes to [10]phenacene and [11]phenacene.

The samples of [10]phenacene and [11]phenacene were synthesized as shown in Fig. 1. Experimental details are described in the Method section. The key step for synthesizing large phenacene molecules is based on the photocyclization of diarylethene^29–35^, known as the Mallory photocyclization, followed by oxidative aromatization to form a phenanthrene-like zigzag benzene array. Diarylethene **5**, which is the precursor to [10]phenacene, was prepared by a Wittig reaction between phosphonium salt **3** and chrysenecarbaldehyde **4** using tetrabutylammonium hydroxide (Bu_4_NOH) as base. The diarylethene **5** was used in the Mallory photocyclization without separation of *E*- and *Z*-isomers; the crude diarylethene **5** was photolyzed at 365 nm in *o*-dichlorobenzene at ca. 150 °C in the presence of a catalytic amount of I_2_ to produce [10]phenacene as an off-white precipitate, which was simply collected by filtration. [11]Phenacene was prepared by a Wittig reaction between bis-phosphonium salt **8** and phenanthrenecarbaldehyde **9**, followed by a two-fold Mallory photocyclization of the resulting diene **10** in boiling *o*-dichlorobenzene. Because precursors **5** and **10** were hardly soluble in common organic solvents, their Mallory photocyclization was carried out at elevated temperatures.

**Figure 2:**
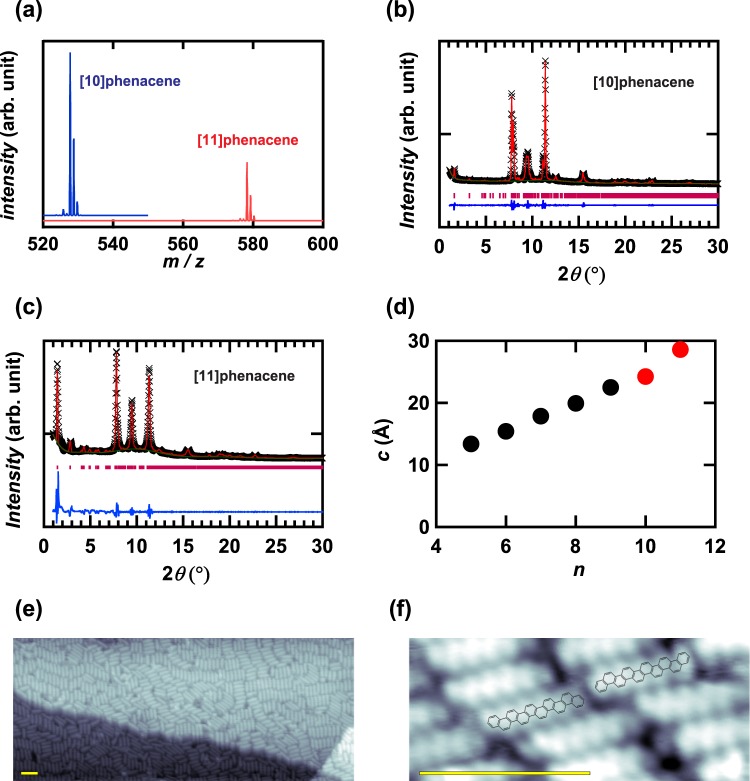
(**a**) MALDI-TOF MS spectra of [10]phenacene and [11]phenacene samples. XRD patterns of powder samples of (**b**) [10]phenacene and (**c**) [11]phenacene. (**d**) *c* – n plots of [n]phenacenes with n = 5–11. The ‘x’ plot and red line refer to the experimental and the calculated patterns, respectively, in (**b**) and (**c**). The red bar and blue line correspond to the position of Bragg reflection and the difference between the experimental and the calculated patterns. (**e**) STM image of 0.9 ML of [10]phenacene molecules deposited on Au substrate, and (**f**) the expanded STM image. The scale indicated by yellow bars ((**e**) and (**f**)) corresponds to 5 nm. The 10-lobes are shown in the STM image.

The [10]phenacene and [11] phenacene molecules were characterized by elemental analyses, their MALDI-TOF MS spectra (Fig. 2a) and powder XRD patterns (Fig. 2b,c). The elemental analyses showed the values of 95.26% for C and 4.36% for H for [10]phenacene where the calculated values are 95.42% for C and 4.58% for H, while the values of 95.07% for C and 4.48% for H for [11]phenacene where the calculated values are 95.47% for C and 4.53% for H. The MS spectra showed peaks at m/z = 527.73, 528.78 and 529.76 for [10]phenacene, and m/z = 578.26, 579.29 and 580.29 for [11]phenacene, respectively, indicating the successful synthesis of these molecules. The splitting of peaks reflects the natural abundance of isotopes of C and H in [10]phenacene (C_42_H_24_) and [11]phenacene (C_46_H_26_). The powder XRD patterns of [10]phenacene and [11]phenacene were analyzed by Le Bail fitting under the space group P2_1_ (monoclinic, No. 4). The lattice constants of [10]phenacene, *a*, *b*, *c*, and *β*, were determined to be 8.5416(7), 6.2281(5), 24.2227(6) Å, and 93.031(4)°, respectively, while those of [11]phenacene were determined to be 9.893(6), 6.097(2), 28.587(5) Å, and 90.83(2)°, respectively.

Here, it is noticed that single crystal XRD measurement could not be achieved because of no success in preparation of single crystals of [10]phenacene and [11]phenacene in this study. In addition, it has been difficult to achieve the single crystal XRD measurement because of the formation of very thin single crystals (plate-shape) for phenacene molecules. The single crystal diffraction measurement may be the future work. Nevertheless, successful synthesis of [10]phenacene and [11]phenacene was evidenced employing the methods described above, as well as those shown below.

The *c* – n plot is shown in Fig. 2d, in which n refers to the number of benzene rings. The *c* value straightforwardly increases with the increase in n, suggesting the elongation of the phenacene framework, additional evidence that molecules of [10]phenacene and [11]phenacene were in fact synthesized. We have obtained more than 30 mg of samples of [10]phenacene and [11]phenacene. This is the first success in obtaining macroscopic amounts of solid samples of the above molecules.

Direct evidence of the molecular structure is given by scanning tunnelling microscope (STM) experiments. [10]phenacene molecules were thermally deposited on an Au(111) surface with ~90% coverage; with less coverage, molecules remain mobile, and the instability renders imaging infeasible. Figure 2e presents a representative large-scale image of [10]phenacene molecules on Au terraces separated by monoatomic Au steps, one running from the left to the right and one located in the bottom right corner. The molecules are closely aggregated in small grains with different orientations, their molecular planes parallel to the Au surface.

As depicted in Fig. 2f, a closer inspection of the single molecule structure reveals a pronounced internal 10-lobe structure, with each lobe corresponding to one of ten benzene rings. This STM image clearly corresponds to a [10]phenacene molecule. Two mirror-symmetric pairs are observed, as shown in Fig. 2f, which is consistent with the result for other [n]phenacene molecules^36^. The growth of the [10]phenacene layer starts with the physisorption of a [10]phenacene molecule on the Au surface, which is characteristic of non-polar molecules deposited on an Au(111) surface. The STM image makes it clear that [10]phenacene molecules are deposited without any thermal damage. This fact is significant because thermal deposition of [10]phenacene and [11]phenacene molecules was used to form thin films as active layers of FET devices. The study on STM of [11]phenacene molecule would be the future work because obtaining the clear STM image requires much time and effort, although it is not shown in this study.

**Figure 3:**
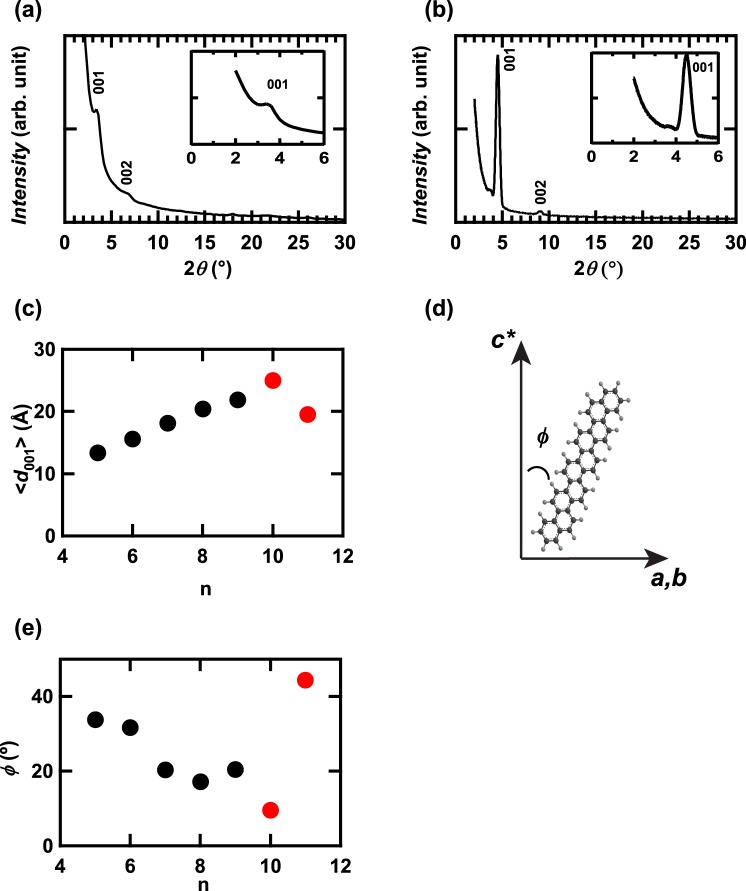
Out-of-plane XRD patterns of thin films of (**a**) [10]phenacene and (**b**) [11]phenacene. (**c**) <*d*_001_> - n plot of [n]phenacene at n = 5–11. The inclined angle, *ϕ*, of an [n]phenacene molecule with respect to c* is schematically shown in (**d**). (**e**) *ϕ* – n plot of [n]phenacene at n = 5–11.

Figure 3a,b show out-of-plane XRD patterns for thin films of [10]phenacene and [11]phenacene, respectively, which were prepared on an SiO_2_/Si substrate by thermal deposition, as described in the Method section. The peaks due to *00l* were observed for these thin films, as seen from Fig. 3a,b. This means that the *ab* planes in both thin films are parallel to the substrate. The value of the inverse absolute reciprocal of *c*, 1/|c^*^|, which corresponds to the distance between *ab*-layer planes, *d*_001_, was determined from each thin film. The average values, <1/|c^*^|> = <*d*_001_>, were evaluated to be 25(1) and 19.5(2) Å for [10]phenacene and [11]phenacene, respectively. The <*d*_001_> − n plot is shown in Fig. 3c. The <*d*_001_> increases straightforwardly with increasing n, up to n = 10, indicating that the angle between the long axis of the molecule and c*, *ϕ*, is almost the same among phenacene molecules with n ≤ 10; the *ϕ* is schematically depicted in Fig. 3d. Actually, as seen from Fig. 3e, the *ϕ* value slowly decreases with n. Then the <*d*_001_> value (=19.5(2) Å) for [11]phenacene is much smaller than that (=25(1) Å) of [10]phenacene despite the larger long axis in [11]phenacene. This suggests two possibilities: (1) [11]phenacene was broken by thermal deposition, (2) The *ϕ* value for [11]phenacene deviates significantly from the trend of *ϕ*-versus-n, in which *ϕ* slowly decreases against n over the range n ≤ 10 (*ϕ* = 10–30°)^28^. The *ϕ* for [10]phenacene was 10°, which is consistent with the above tendency. If the second scenario is the complete explanation, the *ϕ* value of [11]phenacene is estimated to be 45°. This value is inconsistent with the scenario that the strengthening of π-π interaction accompanied by an increase in π-framework must provide progressively smaller *ϕ*^28^. Thus, the reason why the *ϕ* changes drastically at n = 11 remains to be clarified, but the decomposition of [11]phenacene during thermal deposition would be neglected, because of significant experimental evidence as described below.

**Figure 4:**
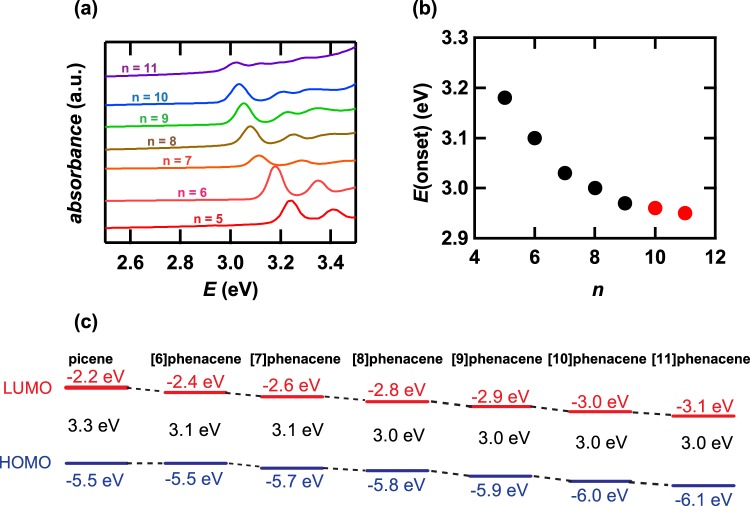
(**a**) UV-VIS absorption spectra of thin films of [n]phenacene with n = 5–11. (**b**) Onset energy (*E*(onset)) - n plot of [n]phenacene with n = 5–11. (**c**) Energy diagram of [n]phenacene (n = 5–11). The numerical value written in black colour refers to the HOMO-LUMO gap.

Figure 4a shows the UV-VIS absorption spectra for thin films of phenacene molecules with n = 5–11. All spectra show a similar pattern, and the first peak at 3.0–3.5 eV shifts systematically to lower energy, which indicates that the first electron excitation energy decreases with n, *i.e*., the band gap becomes narrower against n as n increases from 5 to 11, in the same manner as the band gap versus n at n = 5–9 reported previously^28^. The similarity in UV-VIS absorption spectra for thin films of each phenacene molecule suggests that the [11]phenacene molecule is not broken even in a thin film, *i.e*., by thermal deposition. The onset energy, *E*(onset), of the absorption spectrum, which corresponds to the band gap, is plotted as a function of n (Fig. 4b). The energy for highest occupied molecular orbital (HOMO) was determined for [10]phenacene and [11]phenacene thin films using photoelectron yield spectrum (PYS), and the energy of lowest unoccupied molecular orbital (LUMO) was determined from the energy of HOMO level and band gap (HOMO-LUMO gap). The HOMO and LUMO levels are shown in Fig. 4c, indicating that the HOMO level lowers gradually up to n = 11. This would be also the evidence for no decomposition (or destruction) of sample. Thus, the successful synthesis of [10]phenacene and [11]phenacene led to the systematic elucidation of structural and electronic features of extended phenacene molecules.

### Fabrication and transport characteristics of FET devices using thin films of [10]phenacene and [11]phenacene

No studies on the FET characteristics of [10]phenacene and [11]phenacene FET devices have been reported to date. At the present stage, an FET device using a thin film of the most extended π-network molecule was fabricated using [9]phenacene^28^. Because of difficulty in synthesis, macroscopic amounts of [10]phenacene and [11]phenacene had not yet been obtained, and the FET application of such π-extended molecules was not possible. In this study, FET devices with thin films of [10]phenacene and [11]phenacene were fabricated, in which 400 nm thick SiO_2_ was employed as the gate dielectric. Figure S1a of Supplementary Information shows the FET device structure, and atomic force microscope (AFM) images of thin films of [10]phenacene and [11]phenacene are shown in Fig. S1b and c, respectively, images of the samples that were used for the active layers of FET devices. The rough surface on the thin films of [10]phenacene and [11]phenacene, and the root mean square (RMS) roughness are 6.2 and 9.7 nm, respectively; probably because of the length of the molecule (or large<*d*_001_>).

**Figure 5:**
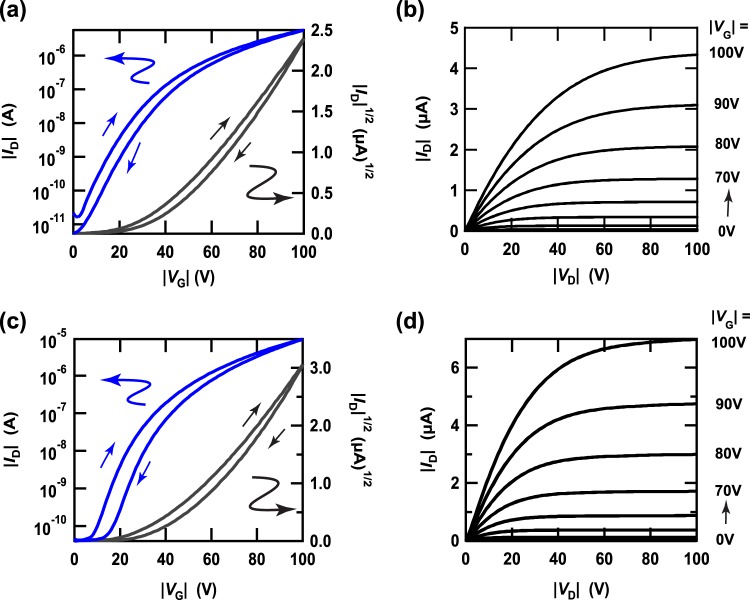
(**a**) Transfer and (**b**) output curves of a [10]phenacene thin-film FET with an SiO_2_ gate dielectric. (**c**) Transfer and (**d**) output curves of an [11]phenacene thin-film FET with an SiO_2_ gate dielectric.

The transfer and output characteristics of a [10]phenacene thin-film FET are shown in Fig. 5a,b, respectively, displaying its p-channel normally-off FET properties. The field-effect mobility, *μ*, absolute threshold voltage, |*V*_th_|, on-off ratio and subthreshold swing, *S*, for the [10]phenacene thin film FET were determined to be 3.70 × 10^−2^ cm^2^ V^−1^ s^−1^, 39.0 V, 3.5 × 10^5^, and 6.5 V decade^−1^, respectively, from the forward transfer curve (Fig. 5a). The averaged values of these parameters were evaluated from five devices to be 3.1(7) × 10^−2^, 40(1) V, 3(2) × 10^5^, and 5(1) V decade^−1^, respectively; the FET parameters of all devices are listed in Table 1. Unfortunately, the averaged *μ* value (<*μ*>) is smaller than that (<*μ*> = 1.2(3) × 10^−1^ cm^2^ V^−1^ s^−1^) of a [9]phenacene thin film FET with SiO_2_ gate dielectric^28^.

**Table 1:** FET parameters of a [10]phenacene thin-film FET with an SiO_2_ gate dielectric.

sample	*μ* (cm^2^V^−1^s^−1^)	|*V*_th_| (V)	ON/OFF	*S* (V/decade)	*L* (μm)	*W* (μm)
#1	3.70 × 10^−2^	39.0	3.5 × 10^5^	6.5	50	500
#2	3.54 × 10^−2^	41.2	7.3 × 10^5^	4.7	50	500
#3	3.64 × 10^−2^	38.6	1.4 × 10^5^	5.9	100	500
#4	2.40 × 10^−2^	40.3	2.5 × 10^5^	2.8	450	1000
#5	2.39 × 10^−2^	40.9	2.1 × 10^5^	4.8	50	500
average	3.1(7) × 10^−2^	40(1)	3(2) × 10^5^	5(1)	—	—

The parameters were determined from the forward transfer curves.

The transfer and output characteristics of an [11]phenacene thin-film FET are shown in Fig. 5c,d, respectively, showing p-channel normally-off FET properties, as in the [10]phenacene thin-film FET. The values of *μ*, |*V*_th_|, on-off ratio and *S* for an [11]phenacene thin-film FET were determined to be 1.18 × 10^−1^ cm^2^ V^−1^ s^−1^, 42.9 V, 3.8 × 10^5^, and 5.5 V decade^−1^, respectively, from the forward transfer curve (Fig. 5c). The averaged values of these parameters were evaluated from five devices to be 1.11(4) × 10^−1^, 48(7) V, 4(2) × 10^5^, and 5(1) V decade^−1^, respectively; the FET parameters of all devices are listed in Table 2. The <*μ*> is almost the same as that (<*μ*> = 1.2(3) × 10^−1^ cm^2^ V^−1^ s^−1^) of a [9]phenacene thin film FET with an SiO_2_ gate dielectric^28^. Finally, we must comment that the FET devices with thin films of [10]phenacene and [11]phenacene operated stably for several months even in air, indicating the stability of these molecules. This is also an advantage of using these molecules as active layers of FET devices.

**Table 2:** FET parameters of an [11]phenacene thin-film FET with an SiO_2_ gate dielectric.

sample	*μ* (cm^2^V^−1^s^−1^)	|*V*_th_| (V)	ON/OFF	*S* (V/decade)	*L* (μm)	*W* (μm)
#1	1.182 × 10^−1^	42.9	3.8 × 10^5^	5.5	50	500
#2	1.086 × 10^−1^	42.7	6.1 × 10^4^	6.1	100	500
#3	1.106 × 10^−1^	47.0	3.4 × 10^5^	3.8	150	500
#4	1.072 × 10^−1^	49.8	2.8 × 10^5^	5.7	200	500
#5	1.093 × 10^−1^	58.8	7.3 × 10^5^	3.5	135	500
average	1.11(4) × 10^−1^	48(7)	4(2) × 10^5^	5(1)	—	—

The parameters were determined from the forward transfer curves.

Here, it should be noted that the high |*V*_th_| and large *S* are observed for the [10]phenacene and [11]phenacene FETs, which are general features of phenacene FETs^19,21,23,26–28^. The most significant factor for the high |*V*_th_| and large *S* may be the density of trap states contained in thin films, *i.e*., trap states of hole in p-channel operation such as phenacene FET^37,38^. Therefore, it is important to reduce the trap states or to effectively fill in the trap states by holes for realizing low |*V*_th_| and small *S*. In the next section, we report low-voltage operation (low |*V*_th_| and small *S*) in [10]phenacene and [11]phenacene FETs by using EDL capacitor, which means the effective filling of the tarp states by holes.

### Fabrication and transport characteristics of EDL FET devices using [10]phenacene and [11]phenacene

**Figure 6:**
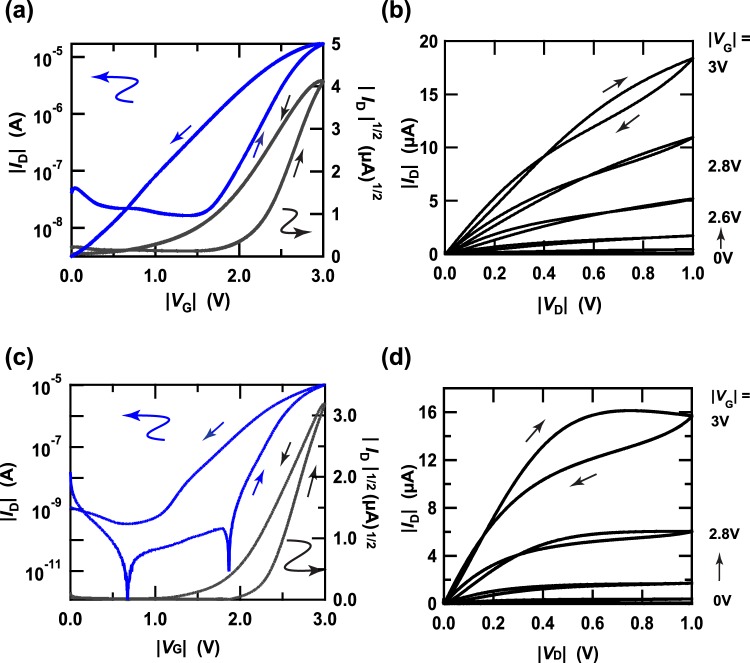
(**a**) Transfer and (**b**) output curves of a [10]phenacene EDL FET. (**c**) Transfer and (**d**) output curves of an [11]phenacene EDL FET.

EDL FET devices with thin films of [10]phenacene and [11]phenacene were fabricated with ionic liquid (butyl-3-methylimidazolium hexafluorophosphate: bmim[PF_6_]) used for the EDL gate dielectric. The active layer was a thin film of [10]phenacene or [11]phenacene. Figure S1d shows the device structure of the EDL FET device, which employed a top gate dielectric and side gate electrode. The transfer and output characteristics of a [10]phenacene EDL FET are shown in Fig. 6a,b, respectively, showing p-channel normally-off FET properties. The operation voltage was too low in comparison with the device with an SiO_2_ gate dielectric (Fig. 5a,b).

The *μ*, |*V*_th_|, on-off ratio and *S* for the [10]phenacene EDL FET were determined to be 4.2 cm^2^ V^−1^ s^−1^, 2.18 V, 1.0 × 10^3^, and 4.0 × 10^−1^ V decade^−1^, respectively, from the forward transfer curve (Fig. 6a). Furthermore, the average values of FET parameters were evaluated from five devices to be 3(1) cm^2^ V^−1^ s^−1^, 2.3(2) V, 2(1) × 10^3^, and 7(5) × 10^−1^ V decade^−1^, respectively; the FET parameters of all devices are listed in Table 3. The FET performance of [10]phenacene EDL FET is quite high.

**Table 3:** FET parameters of a [10]phenacene EDL FET with bmim [PF6]ionic liquid.

sample	*μ* (cm^2^V^−1^s^−1^)	|*V*_th_| (V)	ON/OFF	*S* (V/decade)	*L* (μm)	*W* (μm)
#1	1.4	2.35	1.3 × 10^3^	5.1 × 10^−1^	100	600
#2	3.2	2.61	4.1 × 10^3^	1.9 × 10^−1^	100	214
#3	3.3	2.12	5.2 × 10^2^	1.4	100	450
#4	3.4	2.22	1.5 × 10^3^	8.9 × 10^−1^	100	583
#5	4.2	2.18	1.0 × 10^3^	4.0 × 10^−1^	150	250
average	3(1)	2.3(2)	2(1) × 10^3^	7(5) × 10^−1^	—	—

The parameters for each device (each sample number) was determined from the forward transfer curve.

The transfer and output characteristics of an [11]phenacene EDL FET are shown in Fig. 6c,d, respectively, showing p-channel normally-off FET properties. The operation voltage is also too low in comparison with that using an SiO_2_ gate dielectric (Fig. 5c,d). The *μ*, |*V*_th_|, on-off ratio and *S* for an [11]phenacene EDL FET were determined to be 2.6 cm^2^ V^−1^ s^−1^, 2.30 V, 1.0 × 10^7^, and 1.01 × 10^−1^ V decade^−1^, respectively, from the forward transfer curve (Fig. 6c). Furthermore, the average values of FET parameters were evaluated from five devices to be 1(1) cm^2^ V^−1^ s^−1^, 2.3(1) V, 4(4) × 10^6^, and 1.6(5) × 10^−1^ V decade^−1^, respectively; the FET parameters of all devices are listed in Table 4. The FET performance of an [11]phenacene EDL FET is lower than that of a [10]phenacene EDL FET. To sum up, the value of *μ* is higher in [10]phenacene and [11]phenacene EDL FETs than in the corresponding thin-film FETs with SiO_2_ gate dielectric. The <*μ*> (=3(1) cm^2^ V^−1^ s^−1^) in a [10]phenacene EDL FET is larger than that (=9(2) × 10^−1^ cm^2^ V^−1^ s^−1^)^28^ in a [9]phenacene EDL FET. In addition, these EDL FETs operated stably in Ar-filled glove box for several months, indicating the stability of EDL FET using the thin films of [10]phenacene and [11]phenacene molecules.

**Table 4:** FET parameters of an [11]phenacene EDL FET with bmim [PF6]ionic liquid.

sample	*μ* (cm^2^V^−1^s^−1^)	|*V*_th_| (V)	ON/OFF	*S* (V/decade)	*L* (μm)	*W* (μm)
#1	4.6 × 10^−1^	2.27	2.8 × 10^6^	1.42 × 10^−1^	100	286
#2	1.8	2.41	6.0 × 10^6^	1.36 × 10^−1^	100	232
#3	2.6	2.30	1.0 × 10^7^	1.01 × 10^−1^	100	214
#4	3.1 × 10^−1^	2.11	5.4 × 10^4^	2.29 × 10^−1^	100	563
#5	2.8 × 10^−1^	2.45	1.8 × 10^6^	2.06 × 10^−1^	100	525
average	1(1)	2.3(1)	4(4) × 10^6^	1.6(5) × 10^−1^	—	—

The parameters for each device (each sample number) was determined from the forward transfer curve.

## Discussion

The extended benzene-network molecules, [10]phenacene and [11]phenacene, were synthesized, and these molecules were employed in a thin-film FET. These molecules consist of 10 and 11 fused benzene rings in a repeating W-shaped structure. No attempts at the synthesis of phenacene molecules with 10 and 11 benzene rings have previously been made because of the difficulties in synthesis. We used the ‘Mallory protocol’^26–35^, which consists of the simple repetition of a Wittig reaction followed by photocyclization, for the synthesis, which provided sufficient amounts not only to characterize their crystal structure and electronic properties but also to incorporate in FET devices. Thus, the effectiveness of the Mallory homologation protocol for the synthesis of extended π-network molecules was verified in this study.

In particular, the successful synthesis of phenacene molecules without any functional groups is very significant because the molecular framework may not be distorted. While the π-framework of phenacene molecules with functional groups is distorted^33^, that of phenacene molecules without functional groups is expected to have a planar structure. This simplifies the study of these extended π-network molecules, *i.e*., we can truly pursue the electronic nature of the extended π-network molecules and their utility in electronics without the distraction of side issues such as substituent effects and a distorted π-framework. Therefore, this study provides the first step in the study of the chemistry and physics of extremely extended phenacene molecules and their application in electronics.

The monotonic increase in *c* against n is reasonable because of the elongation of the long axis of the molecule. The decrease in band gap against n (Fig. 4b) is reasonably explained by the elongation of molecules. Admittedly, the band gaps of 2.96 and 2.95 eV for [10]phenacene and [11]phenacene are smaller than that, 3.18 eV, of [5]phenacene (picene) determined in this study. The values of these band gaps are much larger than the 1.84 eV of pentacene^39^, indicating that these molecules are still stable for photo-irradiation in air. This implies that the band gap of an armchair-shaped molecule (*i*.*e*., [n]phenacene) changes little with the elongation of molecule, which should be favorable for device application.

As seen from Fig. 3e, the *ϕ* value becomes smaller with increasing n up to 10, which is explained by the increase in π-π interaction caused by the increase in C atoms (or extension of the π-framework). As suggested in ref.^28^, the van der Waals interaction between phenacene molecules is probably produced by both the π-π interaction through the 2p_z_ orbitals of C and the CH…π interaction, leading to the herringbone stacking of phenacene molecules in the crystals. The π-π interaction should increase because of the extended benzene network, *i.e*., the increase in the number of C atoms as described above. The CH…π interaction should slowly decrease because of the decrease in the ratio of H atoms to C atoms; 0.636 for picene (n = 5) to 0.571 for [10]phenacene (n = 10) and 0.565 for [11]phenacene (n = 11). Consequently, the *ϕ* value should decrease slowly because of the decrease in the above ratio. Admittedly, the *ϕ* gradually decreases up to n = 10, but it rapidly increases at only n = 11 (*ϕ* = 45°), despite the monotonic decrease in the H/C ratio. The reason is still unclear.

Previous reports of the *μ* value versus n in phenacene single-crystal FETs showed a straightforward increase in *μ* up to n = 9, which was reasonably explained by the increase in overlap (π-π interaction) between 2p_z_ orbitals in C atoms^28^. However, the *μ*–n plot (n = 5 to 11) in phenacene thin-film FET showed no significant relationship between *μ* and n^28^. This is probably due to the presence of extrinsic factors other than π-π interaction between phenacene molecules in a thin film, i.e., the crystallinity and presence of defects in thin films may significantly affect the *μ* value. In this study, we could not obtain single crystals of [10]phenacene and [11]phenacene for the construction of a single-crystal FET, so the relation between *μ* and n in a phenacene single-crystal FET could not be obtained.

However, the synthesis of [10]phenacene and [11]phenacene molecules allowed the clarification of the behavior of extremely extended π-network molecules as well as their usability in an FET. Moreover, the [10]phenacene and [11]phenacene EDL FETs showed the averaged *μ* as high as 3(1) cm^2^ V^−1^ s^−1^ and 1(1) cm^2^ V^−1^ s^−1^, which is relatively high. The EDL FETs’ high performance was also observed in [8]phenacene thin film FET^26^, probably owing to the high density of carriers accumulated, which would lead to the lowering of contact resistance between source-drain electrodes and active layer.

Unfortunately, no single-crystal FETs using these molecules was fabricated in this study because of the difficulty of making single crystals suitable for FET devices. Therefore, a straightforward increase in *μ* against n was not recorded in this study, although it is obtained in the single crystal FET with [n]phenacene (n = 5–9). However, p-channel normally-off FET properties have been shown using thin films of these molecules, which does suggest a high potential of [10]phenacene and [11]phenacene for use in practical FET devices. Pursuing the extension of benzene networks in polycyclic aromatic hydrocarbons is currently one of the most important challenges in chemistry and materials science, as well as organic electronics. This work would open the avenue for pursuing chemistry/materials science of extended benzene network molecules (or phenacene molecules), and their application toward organic electronics.

## Methods

### Experimental details of syntheses of [10]phenacene and [11]phenacene

Compounds **1**^34^, **4**^28^, **6**^35^, and **9**^26^ were prepared according to the previously reported procedures. A mixture of 4-methylpicene **1** (398 mg, 1.36 mmol), *N*-bromosuccinimide (NBS, 290 mg, 1.63 mmol), and benzoyl peroxide (BPO, 75%, 22 mg, 0.07 mmol) in chlorobenzene (150 ml) was heated at 110 °C for 22 h. The solvent was removed under reduced pressure and the residue was successively washed with toluene and MeCN. The crude product was recrystallized from *o*-dichlorobenzene to afford 4-(bromomethyl)picene **2** (338 mg, yield of 67%). The obtained compound **2** was heated with triphenylphosphine (286 mg, 1.09 mmol) at 110 °C in *N*, *N*-dimethylformamide (DMF, 30 ml) overnight. The precipitated product was collected and recrystallized from a CHCl_3_-toluene mixture to afford phosphonium salt **3** as pale brown solid (mp > 300 °C, 576 mg, yield of 91%). Physical data are shown in Supplementary Information.

To a solution of phosphonium salt **3** (190 mg, 0.30 mmol) and aldehyde **4** (77 mg, 0.30 mmol) in CH_2_Cl_2_ (30 ml) was added tetrabutylammonium hydroxide (Bu_4_NOH; 1 M in MeOH, *i.e*., 0.50 mmol in 0.5 ml). The solution was stirred at room temperature for 1 h, and the precipitated diarylethene **5** (135 mg, yield of 85%) was collected and successively washed with CH_2_Cl_2_ and toluene. This product was used in the following photoreaction without purification. A solution of diarylethene **5** (42.5 mg, 0.11 mmol) and a small portion of I_2_ in 700 ml of boiling *o*-dichlorobenzene was irradiated with black-light lamps (6 × 15 W) for 15 min. After light irradiation, a precipitate was formed, which was collected and washed with toluene to afford [10]phenacene as off-white plates. This photoreaction was twice repeated to obtain a [10]phenacene powder sample (71 mg, total yield of 71% in two photoreactions).

A mixture of 1, 8-dimethylphenanthrene (1.03 g, 50.0 mmol), NBS (1.78 g, 10.0 mmol), and BPO (162 mg, 0.5 mmol) in CCl_4_ (70 ml) was refluxed for 16 h. The solvent was removed and the residue was washed with MeOH to afford dibromide **7** as colourless solid (mp 180–188 °C, 1220 mg, yield of 67%).

A solution of dibromide **7** (910 mg, 2.5 mmol) and triphenylphosphine (1.44 g, 5.5 mmol) in toluene (50 ml) was refluxed for 18 h. The precipitate formed was collected and washed with toluene to afford bis-phosphonium salt **8** as colourless solid (mp > 280 °C, decomposed, 2160 mg, yield of 97%). Physical data appear in Supplementary Information.

To a solution of bis-phosphonium salt **8** (222 mg, 0.25 mmol) and aldehyde **9** (103 mg, 0.50 mmol) in CH_2_Cl_2_ (40 ml) was added a MeOH solution of tetrabutylammonium hydroxide (1 M, *i.e*., 2.0 mmol for 2.0 ml), and the solution was stirred at room temperature for 14 h. The precipitated product (A) was collected and successively washed with CH_2_Cl_2_ and MeOH. The filtrate was concentrated and the residue (B) was washed with MeOH. The obtained products, A and B, were combined to provide diene **10** (127 mg, yield of 87%) which was used in the following photoreaction without purification.

A boiling *o*-dichlorobenzene solution (700 ml) containing diene **10** (115 mg, 0.20 mmol) and small portion of I_2_ was irradiated with black-light lamps (6 × 15 W) for 1 h. The precipitated product was collected and washed with *o*-dichlorobenzene to afford [11]phenacene as a pale brown solid (92 mg, yield of 79%).

### Characterization of [10]phenacene and [11]phenacene

The MALDI-TOF MS spectrum was measured using a Bruker Autoflex mass spectrometer. The XRD patterns of the thin films of [10]phenacene and [11]phenacene were measured using a RIGAKU SMARTLAB-PRO with Cu Kα source (wavelength of 1.5418 Å), and the XRD patterns of crystalline powders of [10]phenacene and [11]phenacene were measured at BL12B2 of SPring-8 with an X-ray beam of wavelength = 0.68865 Å. The AFM images of thin films of [10]phenacene and [11]phenacene were recorded with a measurement system (SII Nano Technology SPA400). The UV-VIS absorption spectra of thin films of [10]phenacene and [11]phenacene were measured using a UV-VIS spectrometer (JASCO V-670 iRM EX).

STM experiments were performed in an ultra-high vacuum of 10^−10^ Torr using a modified Unisoku USM1300 system. [10]phenacene molecules were thermally deposited at ~530 K at a rate of 0.1 molecular layers per minute onto a clean Au(111) surface. The substrate was kept at room temperature. After sample transfer, the STM imaging was conducted at 77 K with a bias voltage applied to the sample, *i.e*., positive voltages refer to tunnelling into unoccupied sample states.

### Preparation of FET devices using thin films of [10]phenacene and [11]phenacene and measurement of FET characteristics

The surfaces of the SiO_2_ gate dielectrics used for [10]phenacene and [11]phenacene thin-film FETs were treated with 1, 1, 1, 3, 3, 3-hexamethyldisilazane (HMDS) to produce a hydrophobic surface, as is described elsewhere^23^. The thin films were formed on the SiO_2_ gate dielectric by thermal deposition under a vacuum of 10^−6^ Torr, with the substrate kept at room temperature during thermal deposition. Thin films 60 nm thick of [10]phenacene and [11]phenacene were prepared by thermal deposition under a vacuum of 10^−6^ Torr. Au source/drain electrodes 50 nm thick were formed on the thin films by thermal deposition under 10^−6^ Torr. 3 nm thick F_4_TCNQ was inserted between the thin films and the source/drain electrodes to reduce contact resistance. The FET device with an SiO_2_ gate dielectric employed a top-contact and bottom gate structure (Fig. S1a). The above thin films of [10]phenacene and [11]phenacene were used for XRD and AFM measurements.

The EDL polymer sheet was used for the gate dielectric of [10]phenacene and [11]phenacene EDL FETs; the polymer sheet was prepared using [1-butyl-3-methylimidazolium][hexafluorophosphate] (bmim[PF_6_]) and poly(vinylidene fluoride-co-hexafluoropropylene). Details of its synthesis are given in ref.^21^. As described in the Results section, the gate voltage was applied from a side-gate electrode in thin-film EDL FETs, *i.e*., this device employed a side-gate top-contact structure (Fig. S1d).

The FET characteristics were recorded using a semiconductor parameter analyzer (Agilent B1500A) for the FET devices in an Ar-filled glove box. The capacitance per area, *C*_o_, was measured using a precision LCR meter (Agilent E4980A). The *C*_o_ value for evaluation of FET performance was determined by extrapolation of the capacitance measured at 20 Hz–1 kHz to 0 Hz. The *C*_o_ values of SiO_2_ and EDL gate-dielectric (polymer sheet) at 0 Hz were 8.34 nF cm^−2^ and 8.01 μF cm^−2^, respectively. All FET properties were evaluated from the forward transfer curves at saturation regime.

## Supplementary information

Supplementary Information
