# Supplementary material for: Synthesis of the extended phenacene molecules, [10]phenacene and [11]phenacene, and their performance in a field-effect transistor
Source: Sci Rep. 2019 Mar 8;9:4009. doi: 10.1038/s41598-019-39899-4 (PMC6408568; doi:10.1038/s41598-019-39899-4)
Supplement: Supplementary file 1 — Supplementary Information [file 41598_2019_39899_MOESM1_ESM.pdf]

Supplementary Information

Synthesis of the extended phenacene molecules, [10]phenacene and [11]phenacene, and their performance in a field-effect transistor

Hideki Okamoto[1], Shino Hamao[2], Ritsuko Eguchi[2], Hidenori Goto[2], Yasuhiro Takabayashi[1], Paul Yu-Hsiang Yen[3], Luo Uei Liang[3], Chia-Wei Chou[3], Germar Hoffmann[3], Shin Gohda[4], Hisako Sugino[4], Yen-Fa Liao[5], Hirofumi Ishii[5], and Yoshihiro Kubozono[2*]

[1]Department of Chemistry, Okayama University, Okayama 700-8530, Japan

[2]Research Institute for Interdisciplinary Science, Okayama University, Okayama 700-8530, Japan

[3]Department of Physics, National Tsing Hua University, Hsinchu 30013 Taiwan,

[4]NARD Co Ltd., Amagasaki 660-0805, Japan

[5]National Synchrotron Radiation Center, Hsinchu 30076, Taiwan

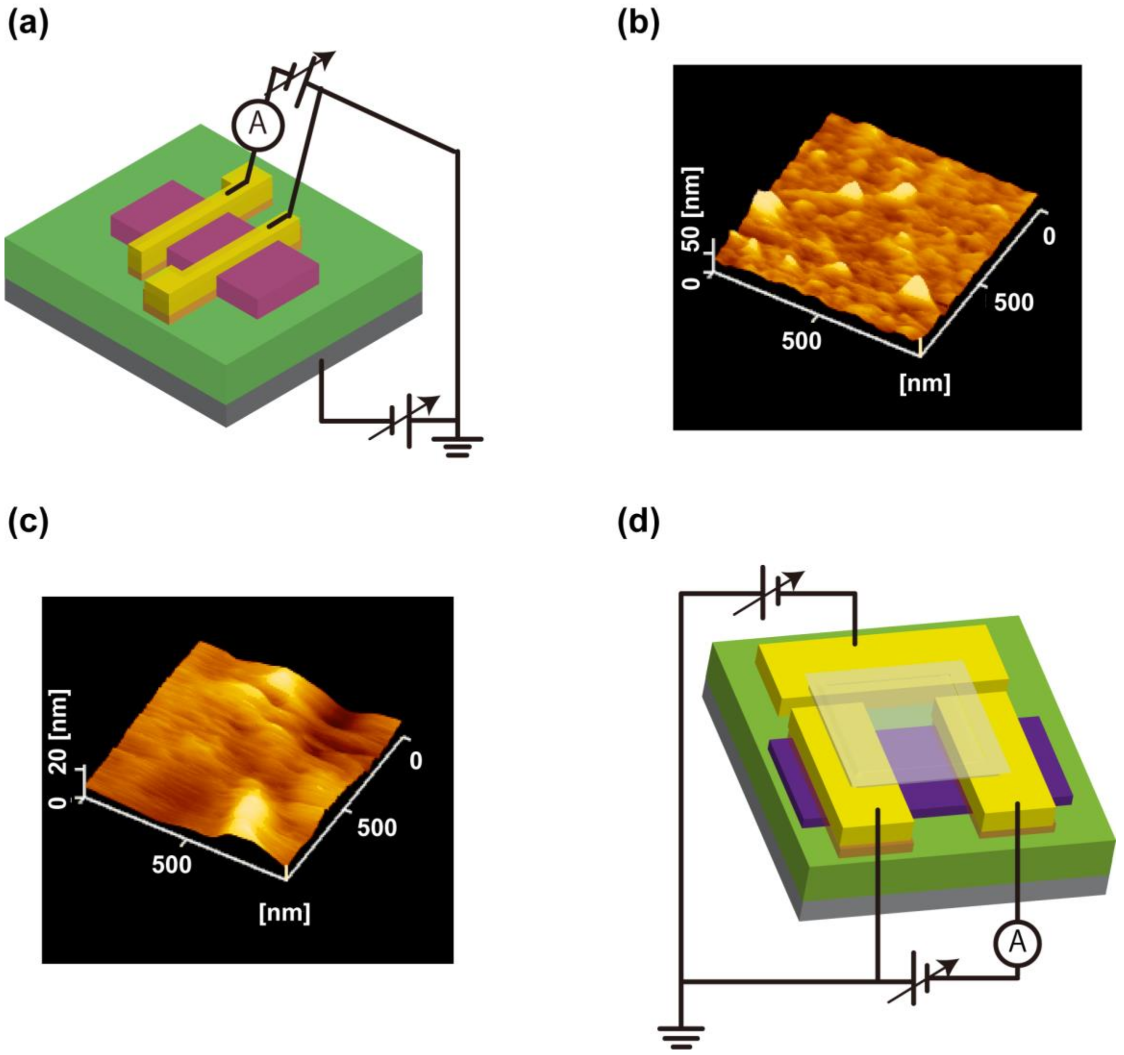

Figure S1. (a) Device structure of [10]phenacene and [11]phenacene thin-film FETs with SiO2 gate dielectric. AFM image of thin films of (b) [10]phenacene and (c) [11]phenacene. (d) Device structure of [10]phenacene and [11]phenacene EDL FETs.

Physical data for novel compounds.

4-(Bromomethyl)picene 2: Off-white plates, mp 283–285 °C (decomp.). ^{1}H NMR (1,1,2,2-tetrachloroethane-d_2, 600 MHz) δ_H = 8.98 (d, 1H, J = 9.4 Hz), 8.96 (d, 1H, J = 9.4 Hz), 8.95 (d, 1H, J = 9.4 Hz), 8.91 (d, 1H, J = 8.3 Hz), 8.87 (d, 1H, J = 8.3 Hz), 8.81 (d, 1H, J = 9.2 Hz), 8.38 (d, 1H, J = 9.3 Hz), 8.08 (d, 1H, J = 9.1 Hz), 8.05 (d, 1H, J = 7.8 Hz), 7.78 (ddd, 1H, J = 8.3, 6.9, 1.2 Hz), 7.74 (d, 1H, J = 7.1 Hz), 7.73–7.68 (m, 2H), 5.12 (s, 2H).

^{13}C NMR (1,1,2,2-tetrachloroethane-d_2, 151 MHz, 60°C) δ_C = 134.0, 132.1, 131.3, 130.4, 129.8, 129.02, 128.90, 128.7, 128.58, 128.54, 128.51, 127.9, 127.1, 126.9, 126.5, 124.7, 123.2, 122.8, 122.6, 122.2, 121.9, 121.6, 32.0. Elemental analysis: Calcd for $C_{23}H_{15}Br$: C, 74.41; H, 4.07. Found: C, 74.47; H, 3.80.

[4-(Picenenyl)methyl]triphenylphosphonium bromide 3: Off-white crystals, mp >300°C. ^{1}H NMR ($CDCl_3$, 600 MHz) δ_H = 8.79 (d, 1H, J = 9.3 Hz), 8.73 (d, 1H, J = 8.2 Hz), 8.71 (d, 1H, J = 9.3 Hz), 8.63 (dd, 1H, J = 8.4, 2.5 Hz), 8.45 (d, 1H, J = 9.0 Hz) 8.18 (d, 1H, J = 9.4 Hz), 7.92 (d, 1H, J = 8.0 Hz), 7.90 (d, 1H, J = 9.2 Hz), 7.77–7.71 (m, 6H), 7.69 (ddd, 1H, J = 8.2, 6.9, 1.4 Hz), 7.66–7.61 (m, 6H),7.52–7.47 (m, 6H), 7.40 (ddd, J = 8.4, 7.6, 1.0 Hz), 6.00 (d, 2H, J_{PH} = 18.0 Hz). ^{13}C NMR (1,1,2,2-tetrachloroethane-d_2, 151 MHz) δ_C = 135.6 ($J_{C\text{-}P}$ = 2.6 Hz), 134.0 ($J_{C\text{-}P}$ = 9.8 Hz), 131.9, 130.9, 130.53 ($J_{C\text{-}P}$ = 4.3 Hz), 130.48 ($J_{C\text{-}P}$ = 12.4 Hz), 130.3 ($J_{C\text{-}P}$ = 6.1 Hz), 130.1, 129.2, 128.8, 128.7, 128.4, 128.1, 128.0, 127.8, 127.3, 127.2, 126.4, 125.5, 124.9, 123.01, 122.95, 122.95, 122.4, 121.6, 121.3, 121.2, 116.7 ($J_{C\text{-}P}$ = 85.5 Hz), 28.5 ($J_{C\text{-}P}$ = 48.6 Hz). HRMS (FAB) Calcd for $C_{41}H_{30}P$: 553.2085. Found: *m/z* 553.2091 [M-Br].

1,8-Bis(bromomethyl)phenanthrene 7: Colorless crystals, mp 186–188°C (decomp.), ^{1}H NMR (600 MHz, $CDCl_3$) δ_H = 8.72 (d, 2H, J = 7.0 Hz), 8.25 (s, 2H), 7.67 (dd, 2H J = 7.1, 1.4 Hz), 7.61 (dd, 2H, J = 8.3, 7.1 Hz), 5.02 (s, 4H). ^{13}C NMR (151 MHz, $CDCl_3$) δ_C 134.1, 131.4, 129.7, 128.8, 126.6, 124.4, 123.0, 31.9. HRMS (FAB) Calcd for $C_{16}H_{12}{}^{79}Br_2$: 363.9286. Found: *m/z* 361.9332 [M^+].

1,8-Bis(triphenylphosphoniomethyl)phenanthrene dibromide 8: Off-white crystals, mp > 285°C (decomp.), ^{1}H NMR (600 MHz, $CDCl_3$) δ_H 8.56 (dd, 2H, J = 7.4, 2.3 Hz), 7.61–7.72 (m, 18H), 7.48–7.56 (m, 14H), 7.42 (t, 2H,J = 7.8 Hz), 6.97 (s, 2H), 5.78 (d, 4H, $J_{P\text{-}H}$ = 13.9 Hz). ^{13}C NMR (151 MHz, $CDCl_3$) δ_C 135.3 ($J_{C\text{-}P}$ = 2.3 Hz), 134.3 ($J_{C\text{-}P}$ = 9.7 Hz), 131.3 ($J_{C\text{-}P}$ = 6.3 Hz), 130.5 ($J_{C\text{-}P}$ = 3.0 Hz), 130.4 ($J_{C\text{-}P}$ = 4.3 Hz), 130.3 ($J_{C\text{-}P}$ = 12.5 Hz), 126.6 ($J_{C\text{-}P}$ = 4.0 Hz), 124.3 ($J_{C\text{-}P}$ = 9.0 Hz), 123.7 ($J_{C\text{-}P}$ = 4.2 Hz), 121.9, 117.4

($J_{C\text{-}P}$ = 85.4 Hz), 27.9 ($J_{C\text{-}P}$ = 47.3 Hz). HRMS (FAB) Calcd for $C_{52}H_{41}P_2$: 727.2684. Found: *m/z* 727.2688 [M-HBr_2].

^{1}H and ^{13}C NMR spectra of novel compounds

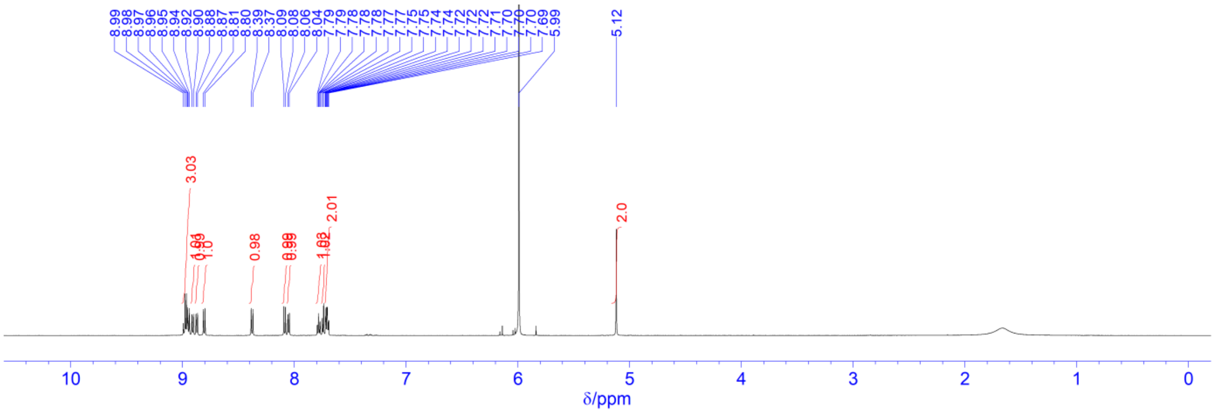

^{1}H NMR spectrum of compound **2** (1,1,2,2-tetrachloroethane-d_2, 600 MHz).

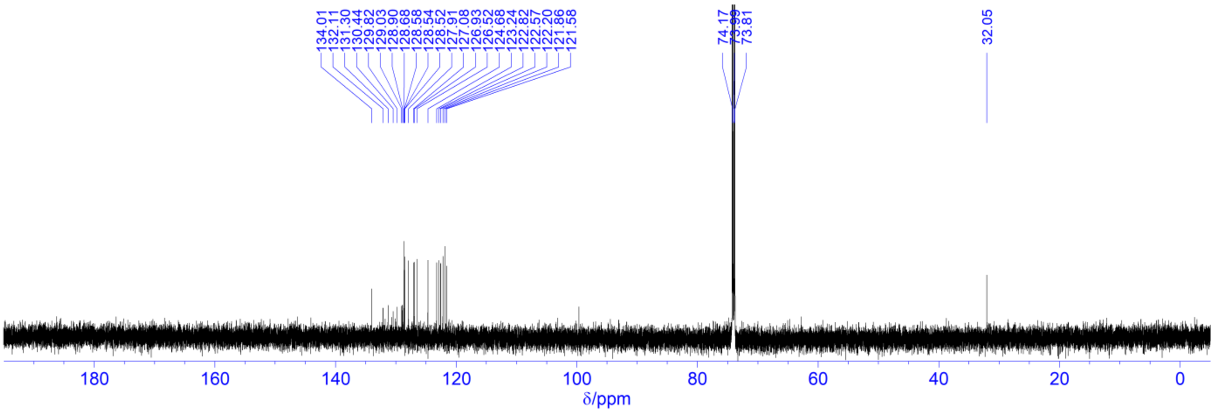

^{13}C NMR spectrum of compound **2** (1,1,2,2-tetrachloroethane-d_2, 151 MHz, 60°C).

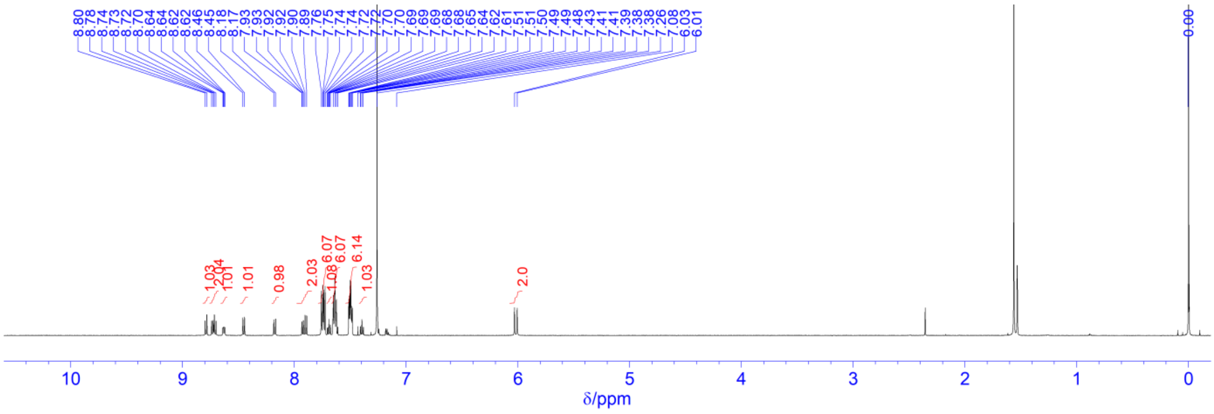

^{1}H NMR spectrum of compound **3** ($CDCl_3$, 600 MHz).

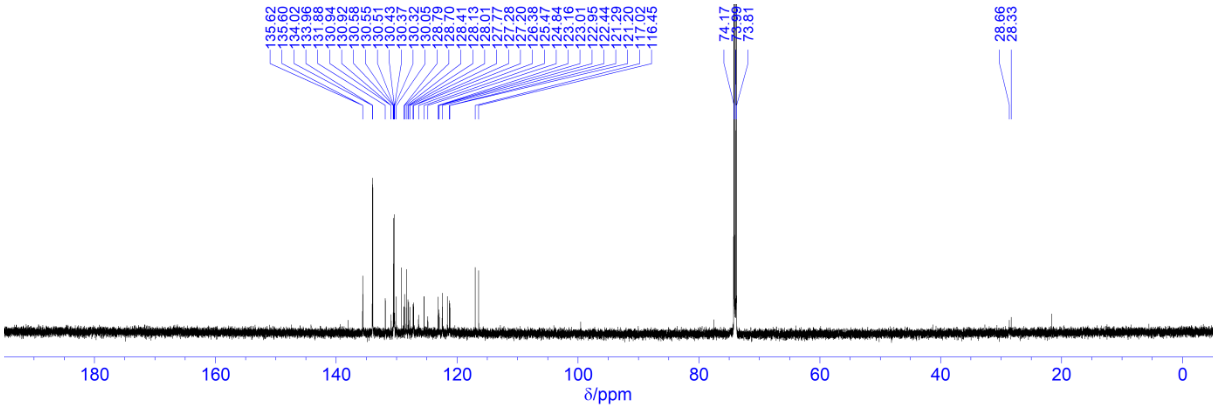

^{13}C NMR spectrum of compound **3** (1,1,2,2-tetrachloroethane-d_2, 151 MHz).

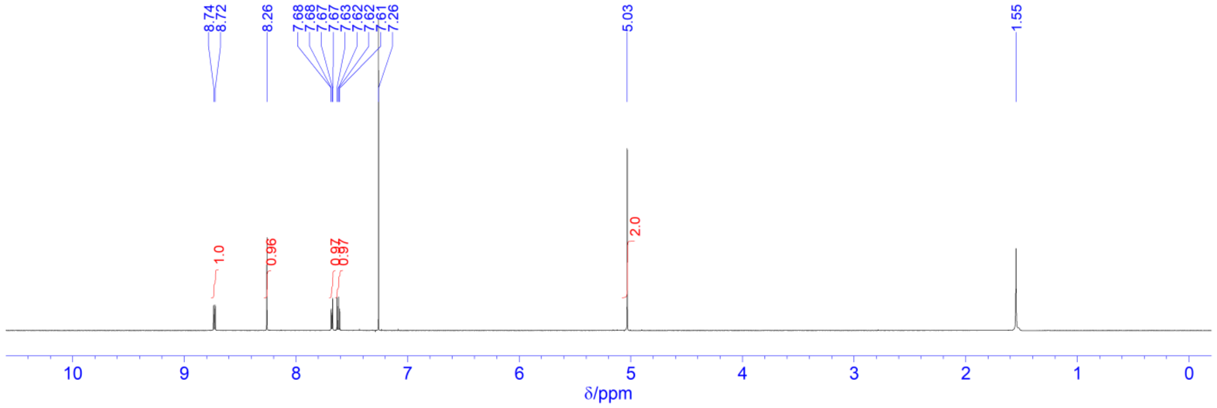

^{1}H NMR spectrum of compound **7** (600 MHz, $CDCl_3$).

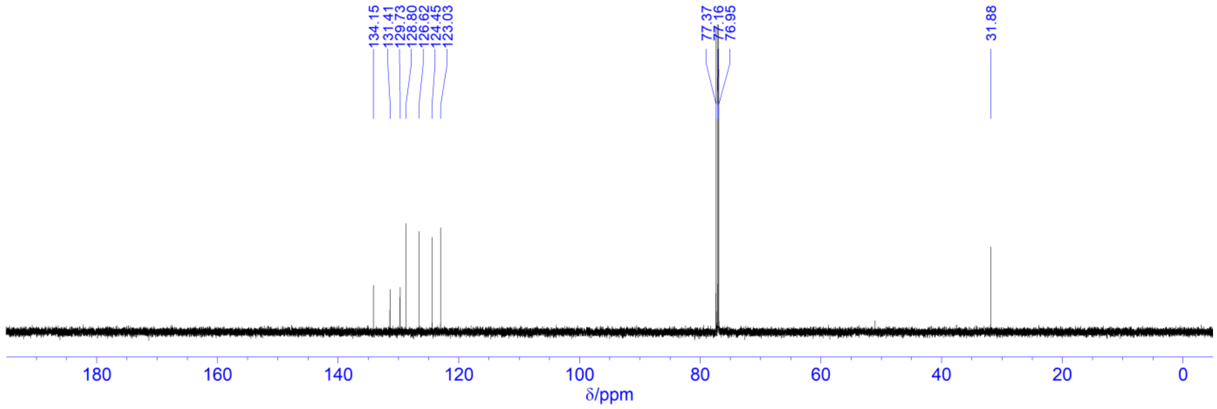

^{13}C NMR spectrum of compound **7** (151 MHz, $CDCl_3$).

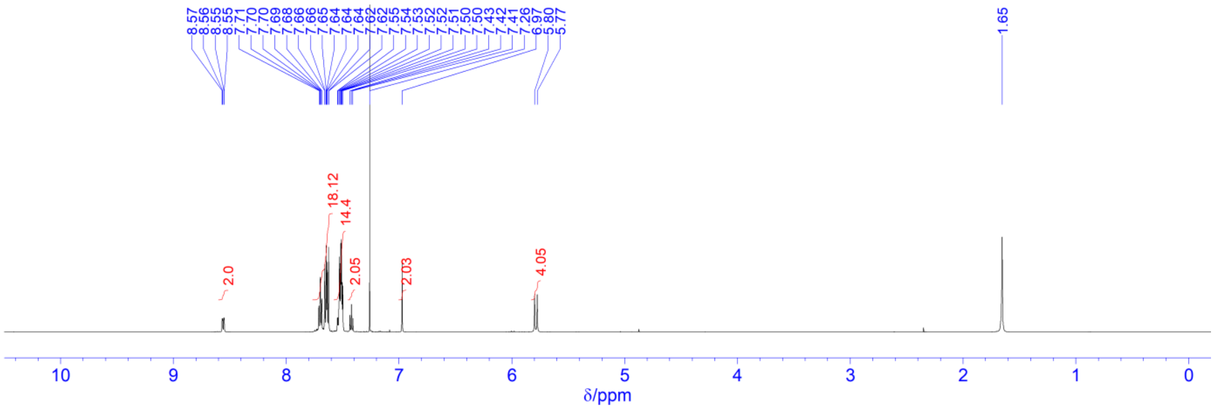

^{1}H NMR spectrum of compound **8** (600 MHz, $CDCl_3$).

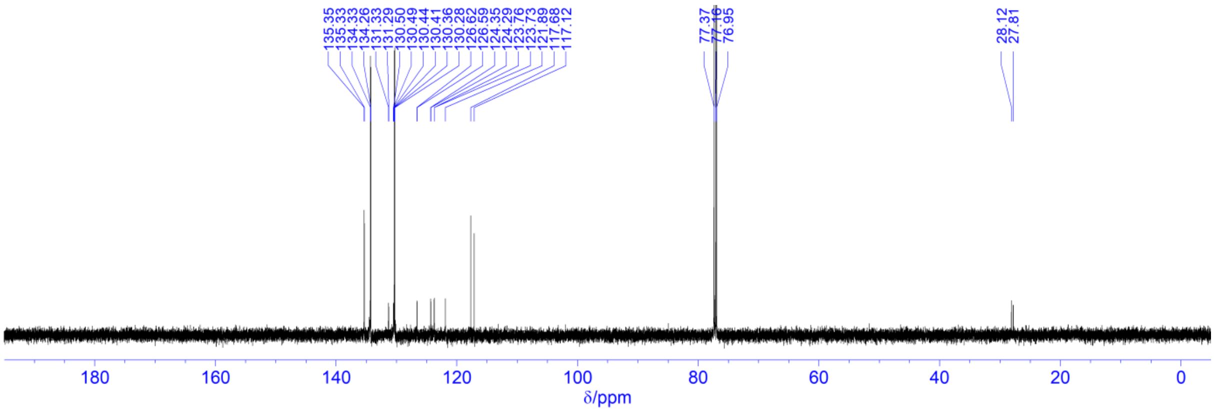

^{13}C NMR spectrum of compound **8**(151 MHz, $CDCl_3$).
